# Interpersonal Communication in Transcultural Nursing Care in India: A Descriptive Qualitative Study

**DOI:** 10.1177/1043659620920693

**Published:** 2020-05-21

**Authors:** Risa Larsen, Elisabeth Mangrio, Karin Persson

**Affiliations:** 1 Malmö University, Malmö, Sweden

**Keywords:** communication, cultural competence, interpreters, language barriers, nursing, quality of care, transcultural nursing

## Abstract

**Introduction:** Good communication is crucial for safe and effective nursing care and is necessary in building interpersonal relationships with patients. The increase of global interactions in health care adds to the necessity of developing culturally competent communication in nursing. The purpose of the study was to gain a deeper understanding of interpersonal communication as experienced by nurses working in culturally diverse hospitals in India. **Method:** A descriptive qualitative method, analyzing 12 semistructured interviews conducted with nurses at two hospitals. **Results:** The study’s themes focus on tools and techniques for working with culturally diverse patients and how to sustain the quality of care in diverse hospital settings. Language resources, language tools, and cultural knowledge were useful aids for nurses when communicating with transcultural patients. It helped the nurses gain confidence and foresee patient needs. **Discussion:** Highlighting transcultural interpersonal communication techniques within nursing offers a safer and more productive practice of nursing care.

## Introduction

In all health care situations, cultural understanding and interpersonal communication are fundamental pillars for care. Interpersonal communication includes empathic understanding, unconditional positive regard, warmth, and genuineness. It creates a relationship where both communicators are equally participating (Encyclopedia.com, n.d.; Pavord & Donnelly, 2015). Cultural understanding and interpersonal communication create the groundwork for nursing goals, education and compliance, and are essential to safe, high-quality nursing care (Schyve, 2007; Travelbee, 2002). The risk of miscommunication, and the potential for resulting damage, increases considerably when patient and caregiver do not speak the same language, or if other cultural or social barriers exist (Kaspar & Reddy, 2017). Nurses working in culturally diverse settings have valuable knowledge and experience in interpersonal cultural communication. Despite this, there is a lack of descriptions by nurse as to their experience and knowledge in communicating with, and caring for, patients with varied cultural backgrounds.

Competence in intercultural communication is complex and multifaceted. It consists of a general understanding, a specific understanding and a positive regard toward other cultures (Wiseman et al., 1989). It is successful when it is appropriate to the specific relationship and effective for all involved parts goals and values (Spitzberg, 2000). Cultural competence in nursing is defined as a continuing and dynamic process with a goal of effectively caring for people with culturally diverse backgrounds by reducing risks and gaining a full understanding of the patient’s emotional needs (Maier-Lorentz, 2008; Sharifi et al., 2019). According to Leininger’s cultural care theory, culturally congruent care can only be provided when the expressions, practices, and patterns of the patient’s culture are known (Leininger & McFarland, 2002). Culturally competent nurses have the necessary sensitivity, knowledge, skill, and proficiency to act with cultural awareness (Sharifi et al., 2019). They have expert knowledge in different cultural practices, cultural humility, cultural assessments, and communication (Sharifi et al., 2019). The ultimate goal of caring for culturally diverse patients is to sustain their health and enable them to effectively cope with death and illness (Leininger & McFarland, 2002; Sharifi et al., 2019). Having specific knowledge of one’s own cultural background as a nurse is also an important factor in creating a humble and sensitive environment that respects the needs of patients, reduces bias, and sustains holistic and competent transcultural nursing care (Maier-Lorentz, 2008; Sharifi et al., 2019).

Language barriers are a crucial hurdle to overcome as they have a negative impact on treatment, safety, and health outcomes for patients. To overcome this barrier, some method of interpretation is often used (van Rosse et al., 2016). Some hospitals have turned to nurses’ ability to speak many languages and use them as means of interpretation (Ali & Johnson, 2017; Repo et al., 2017). Hospitals with a large multilingual patient base tend to use interpreters to bridge the language gap when possible, though the competence and availability of the interpreters vary, and some misinterpretations are considered impossible to avoid (Kaspar & Reddy, 2017). The complexity of medical vocabulary can exacerbate language barriers, making good interpretation a critical part of intercultural communication (Kaspar & Reddy, 2017).

There has been an increase in global interaction between different cultures, making cross-cultural collaboration more frequent (Sharifi et al., 2019). Medical tourism has also started to become an expanding business in many countries around the word, including in India (Health-Tourism.com, 2017). Patients who come to India for medical treatments are a heterogeneous group and differ in ethnic, economic, social, and educational background (Kaspar & Reddy, 2017). A previous study conducted in India shows, however, that nurses are experiencing communication issues when caring for patients from culturally and linguistically diverse backgrounds (Kaspar & Reddy, 2017). Culture-based nursing education as well as additional research in nursing communication is in general needed worldwide to provide nurses with proficient cultural competence in their field (Repo et al., 2017; Sharifi et al., 2019). Thus, the aim of the study was to gain a deeper understanding of interpersonal communication as experienced by nurses working in culturally diverse hospitals in India. Making the findings accessible will enable future nurses and nursing education programs to strengthen their intercultural and interpersonal communication, leading to a safer and more efficient patient-centered care. The aim was to answer the following three research questions:

**Research Question 1:** What characterizes communication while working with culturally diverse patients?
**Research Question 2:** How does cultural competence affect the quality of care in the nurse-patient relationship?
**Research Question 3:** What barriers and enablers affect transcultural interpersonal communication?

## Method

A descriptive qualitative research method was selected for the study, emphasizing interpretation of the participants’ experiences (Sandelowski, 2000). Data were collected through semistructured interviews with open-ended questions. Since the aim of the study was to gather qualitative data about the participants’ experiences regarding intercultural communication, questions were created by carefully analyzing the nature of communication in culturally diverse hospital settings and the aspects that may affect the nurse–patient relationship. The interview questions were tested on the authors prior to interviewing to gain a correct focus and insure no theme was left out (Whiting, 2008). See Table 1, interview guide.

**Table 1.** Sample of Interview Questions.

1. How often do you encounter patients from other cultures different from your own? 2. (a) What other kinds of cultures/nationalities do you encounter in your work? (b) Describe some of these situations. 3. Do you have any previous education within communication, if so what? 4. What is communication to you? 5. How do you communicate with patients who speak a different language from you? 6. (a) How do you find out a patient’s personal desires in combination with their cultural or traditional need within your work? (b) How do you meet these needs? 7. How do you make sure the information passed to patients is received and understood? 8. What would you say are the greatest challenges of transcultural communication? 9. What are your experiences of the effects of miscommunication? 10. (a) What is nursing cultural competence to you? (b) What are your thoughts around the necessity of cultural competence in nursing care? 11. What tools or aids would you need to further support your communication with culturally diverse patients? Further education?

The ethical application was accepted by the Ethical Council at the Faculty for Health and Society, Malmö University on June 16, 2017. Application number HS 2017, nr 59. A document of informed consent was given to and signed by all participants before the start of the interviews, in accordance with the Declaration of Helsinki (World Medical Association, n.d.).

### Sample

Two hospitals were handpicked due to their wide range of international patients. The inclusion criteria were 12 female and male nurses between the age of 20 and 65 years, working with international patients for a minimum of 5 months at one of the two selected hospitals. The origin of the patients included Oman, Saudi Arabia, Sudan, Bangladesh, Nigeria, Europe, the United States, and India. The interviews were conducted in English and the conversations lasted between 21 and 39 minutes.

### Data Collection

Due to the subjectivity of qualitative research, it is important to practice rigor in the research design and method (Cypress, 2017; Lincoln et al., 1985). The authors reinforced the quality of the collected data by employing exact and strict precision in the entire research process. With qualitative research, it is also critical to avoid biases and false interpretations (Cypress, 2017; Lincoln et al., 1985) in order to prevent this two investigators in India, and one at the University of Malmö was involved during the entire research process (Lincoln et al., 1985). By using critical self-reflection throughout the entire research process, and continuously fostering a dialogue, they provided an accurate context of the gathered data, thus avoiding selective observations and personal views (Cypress, 2017; Lincoln et al., 1985). This reflection of bias strengthened the conformability of the study (Lincoln et al., 1985). To ensure effective format for interviewing, a pilot was conducted. The interview questions were not modified from the pilot interview, but one question was added. After careful consideration the pilot interview was included in the final study. The researchers thoroughly reviewed their field experiences and gathered data to find explicit patterns in the different cultures and the concept of transcultural communication. This put the data in context and provided transferability through thick description (Lincoln et al., 1985). Data were collected through persistent observation and after the 12 interviews no new information emerged, thus saturation was considered achieved and of sufficient depth (Guest et al., 2006). By cross-examination of the interpretations and data by both the researchers’, triangulation and peer debriefing were achieved (Lincoln et al., 1985). The interviews were transcribed shortly after each meeting and the authors verified the accuracy of each other’s notes against the recordings to ensure the highest quality of data. These factors established credibility and confirmed the coding of data (Cypress, 2017; Lincoln et al., 1985). The data were externally audited by two researchers not involved in the data collection. They challenged the process of the research and found the conclusions to be supported by the data. This validity of accuracy established dependability of the research (Lincoln et al., 1985). Trustworthiness of the research was thus confirmed through rigor, credibility, dependability, transferability, and conformability of the gathered data (Cypress, 2017; Lincoln et al., 1985).

### Data Analysis

To organize and analyze the gathered data, a thematic content analysis was carried out (Burnard et al., 2008). A careful review of the transcribed words or phrases resulted in an initial coding of the material (Burnard et al., 2008). These initial codes were then sorted into matching categories, which were reduced until the analysis contained 10 categories. Through the process of analysis, every piece of code was applied to only 1 of the 10 categories. Throughout the process, two comprehensive themes were generated. As an example of the analysis procedure an extract of the analysis, with the final set of themes, categories and codes is shown in Table 2.

**Table 2.** Example of Final Themes, Categories, and Codes.

Interview transcript	Code	Category	Theme
**So I know Hindi better and I know English better so I can speak to them, so if the north Indians are coming I can speak Hindi to them. So if we speak in their own language. it will be making it very much comfortable for them also.**	If we speak in the patient’s own language it will make them comfortable.	Language as an important nursing competence	Tools and techniques for working with culturally diverse patients
**But when we both not knowing the particular language, and we want to share something, it is very difficult but definitely I would prefer translator.**	If we don’t speak the same language I prefer to use an interpreter.	The interpreter as a nurse resource	Tools and techniques for working with culturally diverse patients
**Cultural competence or cultural knowledge as a basic thing is very much essential to dealing with patients of different cultures and different traditions, because most of them consider their own culture as a valuable thing.**	Cultural competence is essential because patients consider culture a valuable thing.	Cultural competence as a tool in nursing	How to Sustain quality of care in a diverse hospital setting
**We used to ask different questions like any cultural tools or different cultural differences regarding food. And their bathing pattern, their sleeping pattern we use to enquire.**	Inquiries about cultural preferences regarding food, bathing and sleeping patterns.	Fulfilling the personal and cultural needs of the patient	How to sustain quality of care in a diverse hospital setting

## Results

In the results, two themes emerged, the first focus on the specific tools and techniques the nurses used while working with culturally diverse patients. The first theme included four subthemes: Language as an important nursing competence, The interpreter as a nurse resource, Vehicles for comprehension in health care, and Being prepared in the globalized health care system

The second theme covered aspects on how nurses sustain quality of care in culturally diverse hospitals. Three subthemes were included in this theme: Cultural competence as a tool in nursing, Fulfilling the personal and cultural needs of the patient, and Compassion and comfort in the nurse patient relationship. Language resources and cultural knowledge were found to aid nurses in communicating with transcultural patients. They helped the nurses gain confidence and foresee patient needs.

### Tools and Techniques for Working With Culturally Diverse Patients

This theme refers to the nurse’s personal tools and practical experiences with treating patients with diverse cultural and linguistic backgrounds.

#### Language as an Important Nursing Competence

The participating nurses emphasized the importance of diverse language skills when working with transcultural patient groups, and showed enthusiasm for more in-depth language training. All the nurses in the study spoke at least two languages, English and their mother tongue, Malayalam or Tamil. Several of the nurses also knew additional languages such as Hindi, Arabic, and Malay. They all proposed that language problems and misinterpretations were the main barriers, and one of the biggest challenges when communicating with culturally diverse patients. Most of the patients spoke English, which alleviated many language problems for the nurses since knowledge in English was common among the staff. Problems mainly occurred if the staff was unfamiliar or insufficient in English. Being unable to have an adequate dialogue led to patients becoming worried and uninformed, and jeopardized patient comfort and care.

Some of the announcers and staff here are not much eligible for talking in English, and leads to communication problems. [ . . . ] So most of patients sometimes they worry that nobody’s talking and asking questions. Then leave the patient. Sometimes nurses not at all good at English and other languages, so that’s other different problems. (Nurse #5)

One nurse said patient groups were sometimes divided in accordance with the specific language skills of the nurses working that day, to alleviate information loss, miscommunication, and misinterpretation of complicated medical vocabulary, although it was questionable if this was sufficient for proper translation.

#### The Interpreter as a Nurse Resource

All the interviewed nurses used professional interpreters in their work with culturally diverse patients, but expressed a need for more interpreters to be available at all hours at the hospitals. The interpreters were especially useful in the initial assessment process when the nurse collected vital patient information, and ensured patients knew the routines and practical details of their hospital stay. The nurses said that misunderstandings would quickly be resolved as soon as the interpreter arrived, though any delay might have negative impacts on patient care, such as delayed treatments and concerned patients. The time spent trying to reach an interpreter was also referred to as “a lot of time wasted.”

There is two interpreters but I tried both, and both did not pick my phone, phone was ringing. [ . . . ] Finally they picked up my phone after a few minutes and then they came, they said that she had some pain, and after that we have given pain killers. (Nurse #7)

The nurses said communication problems were avoided if the interpreter was qualified enough, which the majority of interpreters at the hospitals were trusted to be. Occasionally, however, the nurses experienced problems when faced with interpreter incompetence. An incorrect or insufficient translation would directly affect the communication between the patient and the nurse.

Some translators they’re not really keen to help us. Some are very good and some they’re not. They’re just like a doll, whatever you say the doll will talk only. [ . . . ] The translator should not be in that way. They should be more friendly as a friend. Even I don’t know the language, we should talk in nice way to the patient. (Nurse #11)

The nurses expressed that they felt personally responsible in ensuring that patients received correct information even when using an interpreter, and they had different ways of doing so. One option was to observe the patient’s facial expression throughout the translation and afterward ask the patient to repeat what was said. Most of the nurses worked with interpreters who showed a lot of engagement and were involved in patient care and contributed to the medical team. A few nurses, however, said trust issues occasionally occurred when using an interpreter as a mediator. This affected the relationship, unity, and confidentiality between the nurse and patient because of the third party interpreter.

#### Vehicles for Comprehension in Health Care

The interviewed nurses said that starting a conversation with small questions established a foundation of communication. Asking about the patients’ origin, for example, with open-ended questions encouraged culturally competent communication and a relationship of mutual respect, and helped enable patients to feel comfortable enough to speak more freely. The nurses said that this initial gathering of patient information was crucial for fulfilling the patients’ needs during their hospital stay.

Communication is our feelings and our fears explained to other person. To reduce our fear and doubt. . . . Patient communication is very important. Otherwise more more issues. We communicate with the patient more more issues is reduced. (Nurse #6)

All the nurses used nonverbal techniques when communicating with their patients. Actions and gestures were used to relay simple information or ask simple questions. One nurse said, “it was important to be aware of one’s own facial expressions while explaining to the patient.” The nurses used audio–visual aids, flash cards, and written information to help educate and instruct patients. Some nurses argued for the advantages of smartphone apps, Google translate, and computers to aid communication, yet several of the nurses wished for additional creative strategies to enhance their intercultural communication. Manual aids such as written information and educational pamphlets prepared in the language of the patient would be beneficial in the future.

Here we are using charts, visual analog scales, pain scales. But I prefer always advancements. I believe some audio video aids can be used to make patients room oriented. Orientation regarding hospital and orientation regarding rooms. So that the job will be eased for nurses, so we don’t need to explain regarding that again. (Nurse #12)

#### Being Prepared in the Globalized Health Care System

Almost all the participating nurses received communication training as part of their nursing education program. This presented itself as language classes and role-play using various communication techniques. Several of the nurses also received various communication trainings at their workplace, such as language classes and cultural training. All the nurses, however, had a desire to further their knowledge on the subject of culturally competent communication, and some expressed a desire for collaborative workshops with other departments or meetings within their ward to share experiences.

In the nursing education we can include this base of communication with different cultures or different countries, or people in different countries. And also cultures of different countries should be included in the nursing education so it will be very helpful while coming into practice in this situation. (Nurse #2)

Nurses who had worked overseas had gained additional transcultural knowledge and practice in culturally competent communication with foreign patients and colleagues, and encouraged others to go abroad to receive the same understanding.

### How to Sustain Quality of Care in a Diverse Hospital Setting

This theme referred to the awareness and skill required to have interpersonal relationships. It encompassed the cultural knowledge required by nurses to give good and individualized patient-centered care.

#### Cultural Competence as a Tool in Nursing

Due to the globalization of health care, all the interviewed nurses considered knowledge about other cultures important in order to communicate properly with and treat their patients fairly. Cultural competence was not only important, but a basic qualification for the nurses’ work. They defined cultural competence as giving good and safe care while being aware of different cultural or personal needs, no matter what cultural background the patients had. The nurses said that cultural knowledge as a base in the nurse–patient relationship resulted in the ability to work from kindness and understanding which lead to increased patient comfort. Cultural competence, and the caring this presented, increased the nurses’ ability to communicate more effectively and gather relevant information from the patients.

So I think language is not a problem, only how we are approaching the patient. And we should convey the message that “we are here to care for you.” (Nurse #8)

One nurse explained that culture is part of each person, and therefore must be respected. Another emphasized that cultural competence was important because culture is valuable to patients. All the nurses said that the foundation for meeting patient expectations was having knowledge and respect for these personal and cultural differences. They experienced that it was easier to work with patients who had the same cultural background as themselves, and challenging to care for people with different cultural needs or expectations than their own.

When we are working in Kerala we know already this cultures like this and all. . . . We must also know about (other) cultures, because we are mingling with that culture patients and all. So we need more knowledge about their own cultures and all. It’s important for nurses. (Nurse #4)

The participating nurses explained that increased encounters with other cultures helped gain experience and practice in nursing cultural competence. They said that a clear understanding of their own culture was equally important, which helped them understand other cultures better, gave them the ability to be humble in their interactions, and avoiding imposing their own culture on the patients.

#### Fulfilling the Personal and Cultural Needs of the Patient

In order to understand the personal and cultural needs of the patients, the nurses said the first step was to listen carefully. They then asked what the patient needed, what their expectations were or if there was anything else that the patients would like to share. Many patients inquired about places to pray. If the patients did not ask, the nurses would offer help in finding places for religious purposes such as churches, mosques, or temples in or around the hospital. Often a religious representative came to the hospital to perform ceremonies requested by the patients. Nurses also asked about and tried to adapt to, for example, traditions around death, blood transfusions, metal implants, rituals during childbirth, disposal of amputated body parts, or other varying cultural preferences. In some situations, patients wanted to, for example, film the operation, bring religious artifacts into the operation, or hold prayers before procedures. One nurse said that they should not be a hindrance to their patient’s traditions, and tried to support patient inquiries, sometimes requiring help from a supervisor.

I can ask him, ask my superior who will be there. By that time if (family) get reason for ten minutes they can be with the patient, they can continue their tradition. It should not be dislocated because of me. So they can continue their tradition with them, and then they can get out after their cultural programs have been done. (Nurse #3)

According to the nurses, supporting the patients daily cultural needs was especially important. Private rooms and women nurses were encouraged for women patients who were culturally sensitive to exposing their body parts. Sterile hijabs, or other diverse hospital clothing were offered to all patients. Many inquired about specific food items and most often the nurses or nutritionist could provide dietary options.

#### Compassion and Comfort in the Nurse–Patient Relationship

From a compilation of the nurses’ answers, compassion was defined as having empathy, being able to understand and be understood, being considerate and tolerant, and giving comforting care. Comfort meant developing trust between the nurse and the patient, and forming a foundation of trust where the patient felt free to ask any question. Compassion and comfort were very important conditions for mutual communication, listening, understanding, and trust. Especially during shameful or embarrassing moments, the nurses felt that the interpersonal relationship was important to encourage comfort and confidence in the patient. From there the patients could bring up difficult issues or personal concerns. The patients could express fears and concerns which might otherwise not be verbalized and lead to a lower quality of care. The interviewed nurses expressed strong feelings about the importance of treating each patient as their own people, no matter where they came from.

Because patients who come here are not our own people but we treat them as our own people. So when we treat we have to give our knowledge, our love to them as if they feel they are close to us. (Nurse #9)

Some nurses experienced that a smile could give much in terms of patient care, healing, and confidence, especially when other forms of communication were difficult.

All the patients requires care, mainly care. They will be very sick, and if a smile is enough, one smile is enough for their cure. So I believe that all the care is, care, patient care is the most important. (Nurse #1)

The nurses said that giving comfort was the most important skill of the nurse. Even when the nurse only had 5 minutes to speak with a patient, the goal was to make the patient feel comfortable and from there build a trusting relationship.

## Discussion

Intelligible communication between the nurse and patient was considered fundamental to good health care according to all the nurses that were interviewed in this study, as well as in existing literature (Repo et al., 2017). Communication barriers arose when the nurse did not share the same language as the patient or did not have an understanding of the patient’s cultural background (Sharifi et al., 2019). All the participating nurses experienced these intercultural communication difficulties as one of the primary reasons for miscommunication or misunderstanding. The nurses in this study gave examples of both delayed treatment and medication errors related to these communication barriers. In a study about patient safety risks due to language barriers, van Rosse et al. (2016) found that not only were there a number of possible safety risks for patients with low language proficiency, but also that sufficient documentation of these risks was rare. They suggested that standardized record keeping was key to improved communication and enhanced safety of care.

In the present study, the first solution in overcoming language barriers was to call the interpreter, who represented both a vital resource and a source of frustration. This was in line with Kaspar and Reddy (2017) who emphasized the complexity of working with interpreters, translating both between languages and cultures, as well as the problem of availability. Many nurses explained how they sometimes were left stranded without an interpreter and forced to manage patients independently and to the best of their abilities. van Rosse et al. (2016) points to many situations where using an interpreter was inconvenient or unrealistic.

The nurses in the present study emphasized the need to learn the language of the patients. This result was interesting because it indicated how globalization requires new language expertise from professional nurses. They need not only cultural competence and the ability to deliver culturally congruent care but also sufficient language knowledge. The interviewed nurses also viewed language skills as an obvious aspect in their profession and suggested adding language instruction to the nursing curriculum, something several studies also support (Repo et al., 2017; Sharifi et al., 2019). A recent study concludes that both patients and nurses became more comfortable and that patient compliance improved if language barriers are overcome (Ali & Johnson, 2017). Bilingual nurses therefore are, and will continue to be, in high demand (Ali & Johnson, 2017). Some suggestions for improvements involve using technical tools to assist with communication. van Rosse et al. (2016) recommend the use of technological audio-visual aids, such as tablets, as well as providing patients with informed consent forms in their own language.

Effective interpersonal relationships are complex and require empathic understanding, unconditional positive regard, warmth and genuineness (Pavord & Donnelly, 2015). These aspects were clearly woven through all the conversations with the nurses and was reflected in their interpersonal relationships with their patients. The nurses in the study highlighted the importance of kindness, care, and trust in their work. Care and trust are important values in Leininger’s theory on culturally competent care, as well as in Spitzbergs Model of Intercultural Communication Competence. Spitzberg (2000) stated mutual trust increases the communicative relationship. Leininger said that “nurses are expected to get close to people and to establish and maintain intimate caring relationships” (Leininger & McFarland, 2002, pp. 527-561).

The interviewed nurses recognized the importance of cultural competence in their work and as Maier-Lorentz (2008) and Sharifi et al. (2019) highlighted in their studies, they valued cultural assessment, cultural understanding, and communication to give good care. The nurses in this study used their cultural competence to give care which was holistic in nature and culturally specific for the individual patient. This was in line with Leininger’s cultural care theory (Leininger & McFarland, 2002), which states that culturally congruent care can only be provided when the expressions, practices, and patterns of the patient’s culture was known. Like Leininger’s theory of transcultural nursing, the importance of knowing their own cultures, values, and beliefs in order to give good and personal care to their patients was emphasized by the interviewed nurses. The nurse participants expressed that a clear understanding of their own culture gave them a greater ability to respect, become aware of, and satisfy patients with varying cultural needs. They articulated that this was especially important as culture is an influential part of each persons’ personality. These findings describe the nurse participants continuous interactions with culturally diverse populations and could also be described as cultural humility rather than cultural competence (Campinha-Bacote, 2019). And cultural humility is a process of ‘committing to an ongoing relationship with patients, communities, and colleagues’ and it requires humility as individuals continually engage in self-reflection and self-critique (Fisher-Borne et al., 2015). Many of the nurses held their own culture in high regard and said that this gave them a better understanding and a humility in the relationship with patients with other cultural backgrounds, which is also empathized in the recent study by Sharifi et al. (2019). The results indicated some overall distress over the lack of proper preparation and training in transcultural nursing. There was a general request for a stronger emphasis on communication, language, and transcultural education in the nursing education curriculum (Repo et al., 2017; Sharifi et al., 2019).

## Conclusions

Building trust and understanding in an intercultural hospital setting requires culturally aware, goal oriented, and effective communication. This lays the framework for a strong interpersonal nurse–patient relationship and the ability of nurses to recognize each patient as unique. Interpersonal relationships cater to the human being, no matter what culture, traditions, ethnicity, sexuality, age, or gender they hold. In order to truly live and work with integrity, the entire area of health care needs to focus on this holistic interpersonal patient view. As multicultural interactions increase around the world, it is also important to continuously develop effective intercultural communication competence in health care. Since the health care community consistently encounters people of different cultures, highlighting intercultural communication techniques in hospitals and educational systems is crucial for a safe and more productive practice of nursing care. More research needs to be conducted on the subject of interpersonal communication in culturally diverse hospitals since health care is a continuously shifting and growing globalized enterprise.
